# ﻿Species diversity of oysters (Mollusca, Bivalvia) in the intertidal zone of Hainan Island revealed by DNA barcoding analysis

**DOI:** 10.3897/zookeys.1241.139908

**Published:** 2025-06-13

**Authors:** Xin Heng, Fengping Li, Dansheng Xie, Aimin Wang, Chunsheng Liu, Yi Yang

**Affiliations:** 1 School of Marine Biology and Fisheries, Hainan University, Haikou 570228, China Hainan University Haikou China; 2 Sanya Nanfan Research Institute, Hainan University, Sanya 572025, China Hainan University Haikou China

**Keywords:** COI gene, cryptic species, DNA barcoding, Ostreidae, species identification

## Abstract

The family Ostreidae (Mollusca, Bivalvia) is an important component of marine ecosystems. The unique location and marine environment of Hainan Island provide diverse habitats for oysters. However, in recent years, oyster resources of Hainan Island have been under severe threats due to environmental pollution and habitat destruction. To better protect and utilize these biological resources, this study conducted systematic identification of naturally distributed oysters on Hainan Island using DNA barcoding technology. The results revealed the presence of 17 lineages, belonging to 14 species of oysters. The interspecies genetic distances for the COI gene sequences ranged from 10.09% to 31.72%, with notable DNA barcode gaps observed between intra- and interspecies. Additionally, the interspecies genetic distances for the 28S rRNA gene sequences varied between 0.24% and 14.03%. The DNA barcoding analysis indicated the existence of cryptic lineages within *Saccostreacuccullata* (Born, 1778). Furthermore, the study highlighted that *Saccostreamalabonensis* (Faustino, 1932) is the most prevalent and dominant species along the Hainan Island coastline, attributed to its ability to adapt to a wide range of salinity levels. When comparing the species diversity of oysters between the western and eastern coasts of Hainan Island, it was found to be higher on the western coast. This disparity is likely influenced by geographical factors and human activities. Specifically, the western coast, situated in the Beibu Gulf, benefits from relatively stable water quality and numerous river inflows, providing abundant phytoplankton and optimal growth conditions for oyster larvae. Conversely, the eastern coast experiences frequent human activities, such as aquaculture and tourism, which may contribute to the decline in species diversity in this region. Overall, this study enhances our understanding of the species diversity of oysters on Hainan Island and provides scientific evidence that is crucial for the development, protection, and sustainable utilization of these valuable oyster resources.

## ﻿Introduction

The family Ostreidae (Pteriomorphia, Ostreida), which includes bivalve mollusks commonly known as oysters, is globally distributed and plays a crucial role in estuarine ecosystems. Oysters are vital for purifying water and enhancing ecological environments (Searles et al. 2022). Economically, oysters are significant, ranking among the most important species in marine aquaculture (Zarkasi and Nazari 2018). However, their populations are in decline due to climate change, overfishing, habitat destruction, and diseases (Beck et al. 2011). The high variability and plasticity in oyster shell morphology pose challenges for species identification using morphological characteristics, thereby hindering our understanding of oyster diversity (Guo et al. 2018).

The study of oyster taxonomy began with Linnaeus (1758), who first described the genus *Ostrea* (Linnaeus, 1758). Early oyster taxonomy was marked by diverse classification systems based on external morphology. Stenzel (1971) divided Ostreidae into two subfamilies Ostreinae and Lopheinae. A new subfamily, Crassostreinae, was later established by Torigoe (1981) and accepted by Harry (1985). The comprehensive classification among genera within Ostreidae was mostly established by Harry (1985) and revised according to morphological and molecular characteristics (Inaba 2004; Huber 2010; Salvi et al. 2014; Bayne et al. 2017). For example, Salvi et al. (2014) detected considerable genetic divergence between *Crassostrea* Sacco, 1897 and *Saccostrea* Dollfus & Dautzenberg, 1920 and proposed the transfer of the latter genus from Crassostreinae to a new subfamily, Saccostreinae. Molecular evidence also indicated a distance relationship of *Striostrea* Vialov, 1936 and Ostreinae, and the former was elevated to the subfamily Striostreinae (Raith et al. 2016; Salvi and Mariottini 2017). However, the validity of certain genera remains uncertain, and classification at the genus level is still ongoing (Guo et al. 2018).

Molecular markers have also been used in oyster species identification. For example, Klinbunga et al. (2003) developed genetic tools to distinguish three commercially cultured oysters *Crassostreabelcheri* (G.B. Sowerby II, 1871), *C.bilineata* (Röding, 1798), and *Saccostreacuccullata* (Born, 1778), in Thailand based on restriction analysis of 18S rRNA and COI gene fragments. Melo et al. (2010) have also demonstrated the effectiveness of COI gene sequences as molecular markers in molecular identification, phylogenetic analysis, and biogeographical studies of oyster species along the Brazilian coast. Furthermore, Liu et al. (2021) utilized COI gene sequence to identify the dominant oyster species along the coast of Zhejiang, *C.sikamea* (Amemiya, 1928) and *C.angulata* (Lamarck, 1819), laying a foundation for molecular identification and informing conservation and sustainable management of regional oyster populations.

Hainan Island is located at the southernmost of China. The diverse coastal and substrate types, coupled with favorable water quality, create ideal living conditions for mollusks, resulting in a rich abundance of shellfish resources in the intertidal zone (Zhang et al. 2013). Cui et al. (2021) conducted an extensive taxonomic investigation of the genus *Saccostrea* in Hainan Island, leading to a new species *S.mordoides* Cui, Hu, Li, Zhang, Guo & Wang, 2021. Through the collection of wild oyster samples from Hainan Island and other coastal areas, the natural distribution of *C.sikamea* was confirmed, highlighting its widespread presence in this region (Wang et al. 2013; Liu et al. 2024). Biodiversity assessments in the South China Sea further identified *C.dianbaiensis* Xia, Wu, Xiao & Yu, 2014 distributed in Hainan Island, expanding the documented oyster diversity in this biogeographic zone (Xia et al. 2014). Despite these taxonomic advances, research gaps persist regarding spatial distribution patterns and population dynamics of oyster communities on Hainan Island. In recent years, due to overexploitation and environmental pollution, oyster resources on Hainan Island have been continuously declining. Therefore, establishing a comprehensive database of DNA barcodes information is essential to enhance the protection and management of oyster resources (Bucklin et al. 2011; Trivedi et al. 2015; Van Der Pouw Kraan et al. 2024).

## ﻿Materials and methods

### ﻿Sample collection

**Figure 1.**
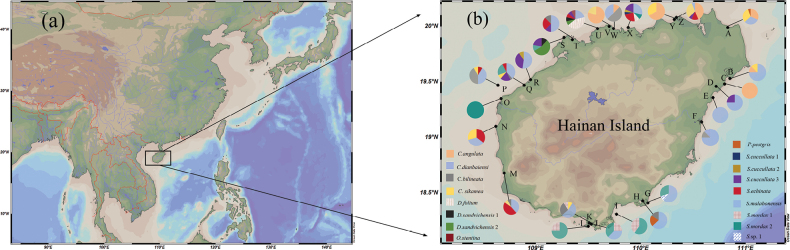
Sampling locations and species distribution. **A.** Proportion of Ostreidae locations of Hainan Island; **B.** Sampling sites.

The oyster samples in this study were collected manually from the intertidal zone of Hainan Island, China, between June 2022 and May 2024. The 26 sampling sites are presented in Fig. 1. The soft tissue was separated from the shell and preserved in 95% ethanol, while shells were deposited for morphological identification. A total of 298 oyster samples were selected for this study, with detailed information provided in Suppl. material 3.

### ﻿DNA Extraction, PCR amplification, and sequencing

Approximately 25–30 mg of the oyster adductor muscle was used for genomic DNA extraction using the Marine Animal DNA Extraction Kit (Tiangen, Beijing, China) according to the manufacturer’s instructions. The quality of DNA was checked by visualizing on 1% agarose gel.

The mitochondrial COI gene fragment was amplified using universal primers (Folmer 1994). The 28S rRNA gene fragment was amplified using primers by Elderkin et al. (2016). For individuals where amplification was unsuccessful, new primers were designed for amplification (Table 1). The polymerase chain reaction (PCR) was conducted in a 20 μL reaction volume, which included 3 μL of template DNA (100 ng/μL), 10 μL of 2× Taq PCR Master MIX II (TIANGEN, China), 0.5 μL of each forward and reverse primer (10 μM), and sterilized distilled water to make up the volume to 20 μL. The PCR conditions were as follows: initial denaturation at 94 °C for 3 min, followed by 35 cycles of denaturation at 94 °C for 30 s, annealing at 48 °C for 30 s, and extension at 72 °C for 45 s, with a final extension at 72 °C for 10 min. The PCR products were checked on a 1.5% agarose gel to confirm the quality and length of the target fragments. Qualified products were sent to Sangon Biotech Company (Shanghai, China) for Sanger sequencing.

**Table 1.** Details of primer sequences used in this study.

Primer name	Primer sequences (5′–3′)	References
LCO1490	GGT CAA CAA ATC ATA AAG ATA TTG G	Folmer et al. 1994
HCO2198	TAA ACT TCA GGG TGA CCA AAA AAT CA	
D23F	GAG AGT TCA AGA GTA CGT G	Elderkin et al. 2016
D6R	CCAGCTATCCTGAGGGAAAC	
HxmlbF	CTACGAATCACCTAGATATTGG	This study
HxmlbR	CTTCAGGGTGCCCGAAAAATCA	

### ﻿DNA barcoding analysis

The COI fragment from both directions were assembled using DNASTAR seqman software and checked manually according to the chromatogram. The assembled COI gene sequences were then submitted to the GenBank database (Suppl. material 3). The COI gene sequences were aligned using the Clustal W program integrated in Mega 6 (Tamura et al. 2013). The base composition of the COI gene sequences was calculated using MEGA, and the genetic distances were computed using the Kimura 2-parameter (K2P) substitution model (Kimura 1980).

Phylogenetic trees were inferred using maximum-likelihood (ML) and Bayesian inference (BI) approaches. ML analyses were conducted in IQ-TREE webserver (Trifinopoulos et al. 2016) with 10,000 ultrafast bootstrap pseudoreplications. BI analyses were performed in MrBayes v. 3.2.7 (Ronquist et al. 2012), employing four parallel Monte Carlo Markov chains (MCMC) for 10 million generations, sampling every 1,000 generations, and discarding the first 25% as burn-in. Two species, *Hyotissasinensis* (Gmelin, 1791) and *H.hyotis* (Linnaeus, 1758) from the family Gryphaeidae, the sister group of Ostreidae, were selected as outgroups.

To establish a robust species delimitation framework, the Assemble Species by Automatic Partitioning (ASAP) method (Puillandre et al. 2021) was utilized. Aligned COI sequences were uploaded to the ASAP web interface (https://bioinfo.mnhn.fr/abi/public/asap), with the Kimura (K80) model selected and all other parameters set to their default values.

## ﻿Results

**Figure 2.**
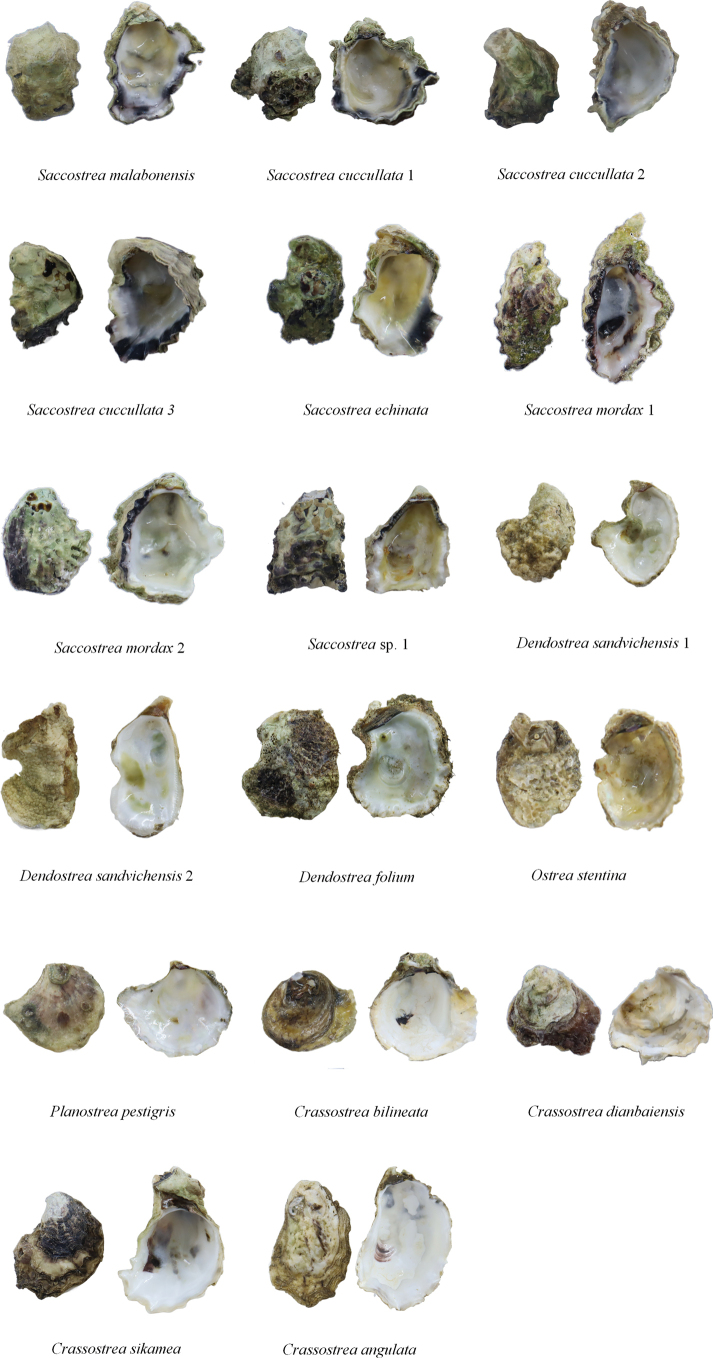
Images of 17 oyster samples collected from Hainan Island.

**Figure 3.**
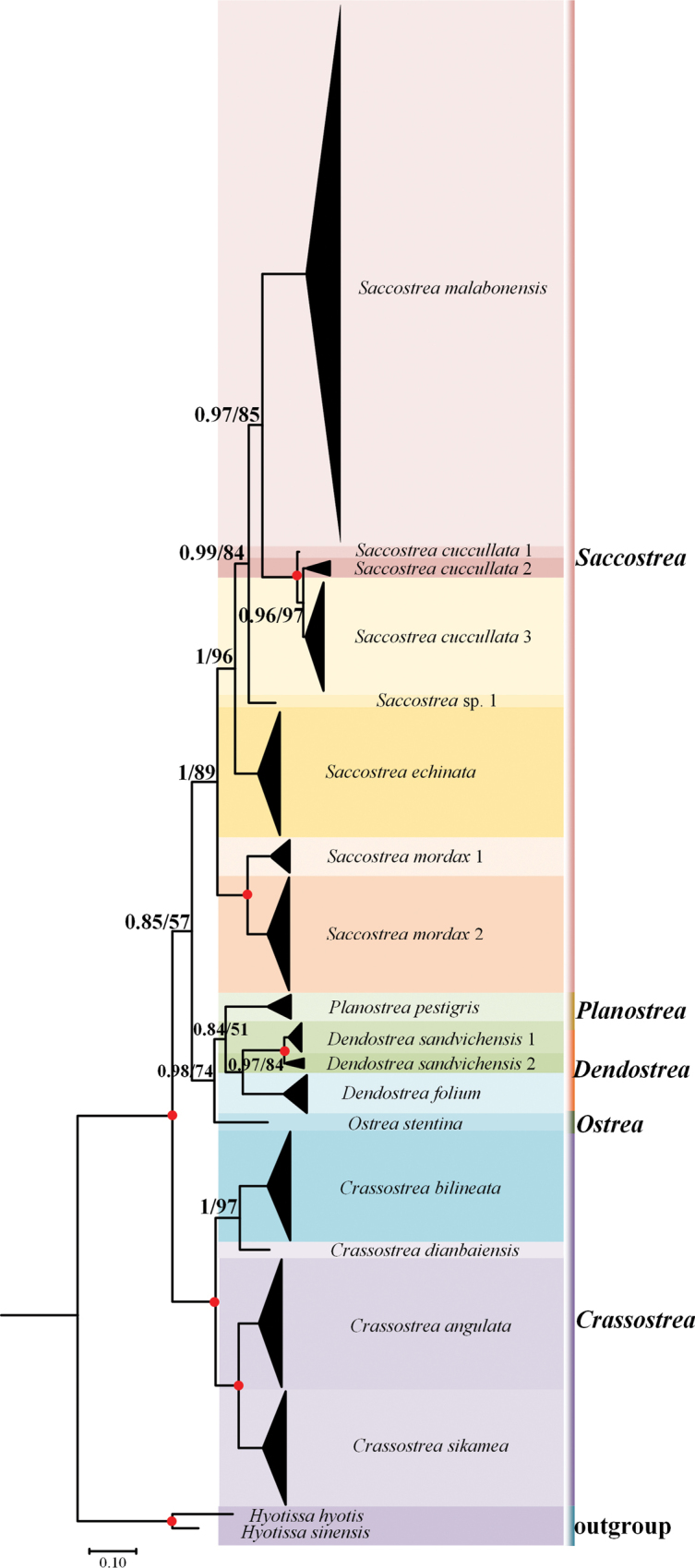
Based on the COI mitochondrial genome sequences, the phylogenetic relationships of the Ostreidae superfamily were reconstructed using *Hyotissahyotis* and *Hyotissasinensis* as outgroups. The first number at each node represents the Bayesian posterior probability (PP), and the second number represents the bootstrap proportion (BP) from ML analysis. Nodes with the highest statistical support (PP = 1; BP = 100) are marked with a solid red circle.

The digital photographs of the oyster species are shown in Fig. 2. A total of 298 COI sequences were obtained through Sanger sequencing, and all sequences were uploaded to GenBank (Suppl. material 3). The aligned COI gene sequences were 587 bp in length. No insertions or deletions were observed in any of the sequences. The average nucleotide composition across all COI gene fragments was 38.9% (T), 17.6% (C), 22.9% (A), and 20.7% (G). The phylogenetic trees generated by the ML and BI methods exhibited a consistent topology (Fig. 3). All individuals formed 17 lineages belonging to 13 species, including *C.angulata*, *C.bilineata*, *C.dianbaiensis*, *C.sikamea*, *Dendostreafolium* (Linnaeus, 1758), *D.sandvichensis* (G. B. Sowerby II, 1871) lineages 1 and 2, *Ostreastentina* Payraudeau, 1826, *Planostreapestigris* (Hanley, 1846), *Saccostreacuccullata* lineages 1, 2 and 3, *S.echinata* (Quoy & Gaimard, 1835), *S.malabonensis* (Faustino, 1932), *S.mordax* (Gould, 1850) lineages 1 and 2, and an unidentified species of *Saccostrea*. The pairwise genetic distances based on COI gene sequences ranged from 1.78% to 31.72% (Table 2). The maximum interspecies genetic distance was observed between *C.bilineata* and *S.cuccullata* lineage 1 (31.72%).

**Table 2.** Mean inter-species and mean intra-species genetic distance (K2P percent) based on COI gene of Ostreidae.

		1	2	3	4	5	6	7	8	9	10	11	12	13	14	15	16	17
1	*Crassostreaangulata*																	
2	*Crassostreabilineata*	18.23																
3	*Crassostreadianbaiensis*	18.42	12.77															
4	*Crassostreasikamea*	10.28	19.16	18.41														
5	*Dendostreafolium*	30.83	28.28	30.46	28.29													
6	*Dendostreasandvichensis* 1	28.80	31.66	29.58	30.45	17.4												
7	*Dendostreasandvichensis* 2	28.97	30.73	29.67	29.29	17.54	2.11											
8	*Ostreastentina*	27.23	26.65	29.75	29.29	20.83	20.62	21.18										
9	*Planostreapestigris*	28.78	25.60	28.68	28.86	17.68	20.87	20.03	21.15									
10	*Saccostreacuccullata* 1	25.25	31.72	30.85	29.42	28.73	26.00	26.7	27.19	25.73								
11	*Saccostreacuccullata* 2	25.45	30.69	31.23	30.07	30.02	26.77	27.52	26.09	26.90	2.56							
12	*Saccostreacuccullata* 3	24.76	30.95	30.35	29.95	29.21	26.78	27.5	27.79	26.77	2.38	1.78						
13	*Saccostreaechinata*	28.32	26.81	26.62	27.14	23.56	22.79	21.48	24.50	24.33	16.03	16.88	17.46					
14	*Saccostreamalabonensis*	29.11	26.42	28.91	30.31	27.04	24.51	23.10	28.43	24.93	16.50	17.64	17.39	17.57				
15	*Saccostreamordax* 1	29.30	26.33	27.77	29.55	23.48	24.32	23.30	25.08	24.12	20.78	22.38	22.69	17.03	20.39			
16	*Saccostreamordax* 2	29.25	26.37	30.49	29.95	23.97	24.42	24.20	24.95	23.82	19.49	21.10	21.63	16.79	19.98	10.09		
17	*Saccostrea* sp. 1	28.35	26.86	26.87	29.95	26.29	23.11	22.93	26.22	23.81	15.42	16.46	15.62	13.29	15.83	20.76	18.63	–

A total of 80 28S rRNA gene sequences were obtained for the 17 lineages identified by COI fragment. The aligned sequences were 714 bp in length. The average nucleotide composition was 20.0% (T), 28.0% (C), 17.8% (A), and 34.3% (G). The pairwise genetic distances within the 17 oyster lineages are presented in Table 3. The results showed that the interspecies genetic distances based on the 28S rRNA gene ranged from 0.07% to 14.03%. The greatest interspecies genetic distance was observed between *C.dianbaiensis* and *D.sandvichensis* lineage 1 (14.03%), while the smallest was between *D.sandvichensis* lineages 1 and 2 (0.07%). The phylogenetic trees based on the 28S rRNA gene was shown in Suppl. material 1. However, *S.malabonensis*, *S.cuccullata* lineages 1–3, *S.echinata* and *S.* sp. were not well distinguished, with relatively low bootstrap support in the nuclear gene tree.

**Table 3.** Mean inter-species and mean intra-species genetic distance (K2P percent) based on 28S rRNA gene of Ostreidea.

		1	2	3	4	5	6	7	8	9	10	11	12	13	14	15	16	17
1	*Crassostreaangulata*																	
2	*Crassostreabilineata*	1.89																
3	*Crassostreadianbaiensis*	2.40	1.72															
4	*Crassostreasikamea*	0.49	1.88	2.33														
5	*Dendostreafolium*	10.69	11.83	12.07	11.52													
6	*Dendostreasandvichensis* 1	12.33	13.42	14.03	13.11	2.87												
7	*Dendostreasandvichensis* 2	12.10	13.18	13.74	12.93	2.82	0.07											
8	*Ostreastentina*	9.08	9.59	10.16	9.65	6.50	7.65	7.49										
9	*Planostreapestigris*	9.82	10.81	10.96	10.63	8.31	9.88	9.84	7.00									
10	*Saccostreacuccullata* 1	7.14	7.44	8.06	7.88	8.97	9.95	9.79	7.56	8.27								
11	*Saccostreacuccullata* 2	8.76	9.06	10.22	9.54	9.91	11.40	11.26	9.03	10.09	2.61							
12	*Saccostreacuccullata* 3	6.99	7.26	8.24	7.68	8.75	9.69	9.57	7.34	8.36	0.81	2.09						
13	*Saccostreaechinata*	7.14	7.49	8.38	7.88	8.71	9.77	9.62	7.4	8.37	0.81	1.99	0.57					
14	*Saccostreamalabonensis*	7.43	7.74	8.75	8.07	8.78	9.74	9.67	7.34	8.46	1.03	2.11	0.55	0.68				
15	*Saccostreamordax* 1	9.18	9.60	9.92	10.02	10.94	11.36	11.3	10.15	11.33	4.54	6.41	4.67	4.60	5.00			
16	*Saccostreamordax* 2	9.20	9.71	10.05	10.15	9.98	10.56	10.46	9.31	10.45	4.06	6.18	4.37	4.27	4.72	0.24		
17	*Saccostrea* sp. 1	4.95	4.79	5.77	5.80	8.37	9.34	9.15	7.36	7.63	1.20	1.01	0.73	0.76	0.88	4.66	4.10	–

The ASAP analysis using COI gene presents 10 partitioning schemes, among which four marked with asterisks were identified as the optimal solutions (Table 4, Suppl. material 2). Among the four optimal solutions, setting the threshold distance to 0.016443 resulted in the recognition of 17 operational taxonomic units (OTUs). This outcome was consistent with the phylogenetic relationships inferred from both the ML and BI trees, further validating their accuracy and reliability.

**Table 4.** Output results obtained with the ASAP method: list of the 10 delimitations with the best ASAP scores.

Nb of subsets	Asap-score	P-val (rank)	W (rank)	Treshold dist.
14	1.50	1.00e-05 (1)	2.69e-04 (2)	0.101666
14	2.50	2.50e-04 (4)	4.01e-04 (1)	0.117710
14	3.00	5.00e-05 (2)	1.29e-04 (4)	0.087768
14	3.00	2.00e-04 (3)	1.65e-04 (3)	0.055915
14	6.50	8.28e-04 (6)	7.18e-05 (7)	0.132054
^*^15	7.00	9.39e-02 (9)	8.56e-05 (5)	0.022919
^*^17	8.00	5.62e-02 (8)	2.07e-05 (8)	0.016443
14	9.00	2.30e-02 (7)	1.44e-05 (11)	0.146539
^*^19	11.00	2.10e-01 (10)	1.29e-05 (12)	0.011942
^*^18	11.00	3.47e-01 (12)	1.67e-05 (10)	0.015006

## ﻿Discussion

### ﻿DNA barcoding analysis

Currently, over 1600 oyster COI sequences have been published in GenBank (https://www.ncbi.nlm.nih.gov/) covering more than 50 oyster species and providing a substantial data foundation for species identification. In this study, oyster sequences obtained through sequencing were compared with publicly available sequences to facilitate species identification. The use of the COI gene as a DNA barcode for species identification, particularly for mollusks, has gained considerable attention (Sun et al. 2012; Ran et al. 2020).

Genetic distance analyses of COI sequences demonstrated distinct non-overlapping ranges between intraspecific and interspecific genetic divergence within oyster taxa, revealing a clear “DNA barcode gap” that supports its utility for robust species-level identification​(Liu et al. 2011). In contrast, 28S rRNA sequences exhibited insufficient resolution for precise species discrimination in oysters, though they retained value for preliminary genus-level classification​(Ó Foighil and Taylor 2000). However, for more refined classification requirements, it may be necessary to combine other molecular markers or technical approaches for comprehensive analysis (Bucklin et al. 2011).

### ﻿Species diversity of oysters along the coast of Hainan Island

Through COI genetic distance and phylogenetic trees indicated 17 lineages of oysters from five genera along the coast of Hainan Island. As the dominant oyster species in Hainan Island, *S.malabonensis* accounted for 38.26% of the total specimens and found at 19 sampling sites, primarily growing in the mid- to low intertidal zones and shallow subtidal zones (Siringan et al. 2016). This species exhibits the adaptability to a wide range of salinity (range:] 12–34‰; Suppl. material 3), which may be attributed to its broad distribution. Notably, *C.dianbaiensis*, first reported in Hainan at Qinglan Port, Wenchang (Xia et al. 2014), was newly documented at the Wanquan River estuary (Fig. 1), extending its known biogeographic range. Additionally, this study provides the first record of *O.stentina* in Hainan. Previously known from eastern Atlantic–Mediterranean regions (Lapègue et al. 2006) and Northwest Pacific populations in China (Taiwan Island) and Japan (Hamaguchi et al. 2017), its discovery highlights Hainan Island as a biogeographic transition zone for oyster diversity.

The DNA barcoding analysis revealed several potential cryptic lineages within Ostreidae. For *S.cuccullata* lineages 2 and 3, the intra-lineage genetic distances are 0.19% and 0.18%, respectively, with an inter-lineage genetic distance of 1.78%. The genetic distances between these lineages are all below the 2% species delimitation threshold defined by Hebert et al. (2003). For *D.sandvichensis* lineages 1 and 2, the intraspecific genetic distances are 0.36% and 0.33%, respectively, with an inter-lineage genetic distance of 2.11%. Although the inter-lineage genetic distance exceeds the 2.0% threshold, it is not greater than 10 times the intraspecific genetic distances (Hebert et al. 2003). The genetic distance between *S.mordax* lineages 1 and 2 reaches 10.09%. The two lineages in the present study corresponds to *S.mordax* A and C in Cui et al. (2021). Our data support the designation of *S.mordax* lineage 2 as a distinct species, as proposed by Cui et al. (2021). The *S.cuccullata* lineage 1 comprises a solitary individual, with a genetic divergence exceeding 2% from the other two lineages. Although the ASAP method also identified these OTUs, it is difficult to determine whether it is an independent species. Therefore, this study takes a more conservative approach by considering it as a potential cryptic lineage.

### ﻿Factors influencing species diversity of oysters in Hainan Island

The results indicated a certain degree of species diversity difference between the east and west coasts of Hainan Island, which is speculated to be related to ocean current activity (Lal et al. 2017). The Beibu Gulf warm current flows along the western and northern coasts of Hainan Island. On the one hand, the water transported by the Beibu Gulf current typically maintains relatively stable temperatures, which is crucial for oyster growth (Mao et al. 2006). On the other hand, the western coast of Hainan Island boasts numerous rivers discharging into the sea. These estuarine zones are abundant with phytoplankton, serving as a primary food source for oysters. Furthermore, the Beibu Gulf current transports ample phytoplankton resources, which can sustain the high-density growth of oysters and contribute to enhanced species diversity in the area (Lai et al. 2014; Xu et al. 2023).

The heterogeneity of species diversity between the east and west coasts of Hainan Island may also be significantly influenced by human activities. Anthropogenic factors, including aquaculture, port construction, and tourism development, contribute to habitat alterations that impact oyster populations. A study by Shi et al. (2024) indicated that in certain regions along the east coast of Hainan Island with intensive aquaculture activities, such as Huiwen, the accumulation of organic matter and residual chemicals in aquaculture wastewater discharged into the sea leads to water quality degradation, ultimately increasing oyster mortality rates. It is crucial to strengthen marine ecological protection, restore natural habitats, and implement sustainable strategies for the conservation and management of oyster populations.

## ﻿Additional information

### Author ORCIDs

Yi Yang https://orcid.org/0000-0002-8911-8501

### Data availability

All of the data that support the findings of this study are available in the main text or Supplementary Information.

## Supplementary materials

Supplementary material 1 The phylogenetic relationships of the Ostreidae superfamily Data type tif Explanation note Based on the 28S nuclear gene nucleotide sequences, the phylogenetic relationships of the Ostreidae superfamily were reconstructed using *Hyotissahyotis* and *Hyotissasinensis* as outgroups. The first number at each node represents the Bayesian posterior probability (PP), and the second number represents the bootstrap proportion (BP) from the ML analysis. Nodes with the highest statistical support (PP = 1; BP = 100) are marked with a solid red circle. https://binary.pensoft.net/file/1352836This dataset is made available under the Open Database License (http://opendatacommons.org/licenses/odbl/1.0/). The Open Database License (ODbL) is a license agreement intended to allow users to freely share, modify, and use this Dataset while maintaining this same freedom for others, provided that the original source and author(s) are credited.Xin Heng, Fengping Li, Dansheng Xie, Aimin Wang, Chunsheng Liu, Yi Yang

Supplementary material 2 Output results obtained with the ASAP method Data type pdf Explanation note Output results obtained with the ASAP method: extract graphical output showing the different delimitations together with the ultrametric clustering tree; each column represents a partition, and the colors represent the OTUs. https://binary.pensoft.net/file/1352837This dataset is made available under the Open Database License (http://opendatacommons.org/licenses/odbl/1.0/). The Open Database License (ODbL) is a license agreement intended to allow users to freely share, modify, and use this Dataset while maintaining this same freedom for others, provided that the original source and author(s) are credited.Xin Heng, Fengping Li, Dansheng Xie, Aimin Wang, Chunsheng Liu, Yi Yang

Supplementary material 3 The details of Ostreidae specimens used in this study Data type docx https://binary.pensoft.net/file/1352838This dataset is made available under the Open Database License (http://opendatacommons.org/licenses/odbl/1.0/). The Open Database License (ODbL) is a license agreement intended to allow users to freely share, modify, and use this Dataset while maintaining this same freedom for others, provided that the original source and author(s) are credited.Xin Heng, Fengping Li, Dansheng Xie, Aimin Wang, Chunsheng Liu, Yi Yang
