# Supplementary figures and images for: ﻿Species diversity of oysters (Mollusca, Bivalvia) in the intertidal zone of Hainan Island revealed by DNA barcoding analysis
Source: Zookeys. 2025 Jun 13;1241:247–60. doi: 10.3897/zookeys.1241.139908 (PMC12238981; doi:10.3897/zookeys.1241.139908)

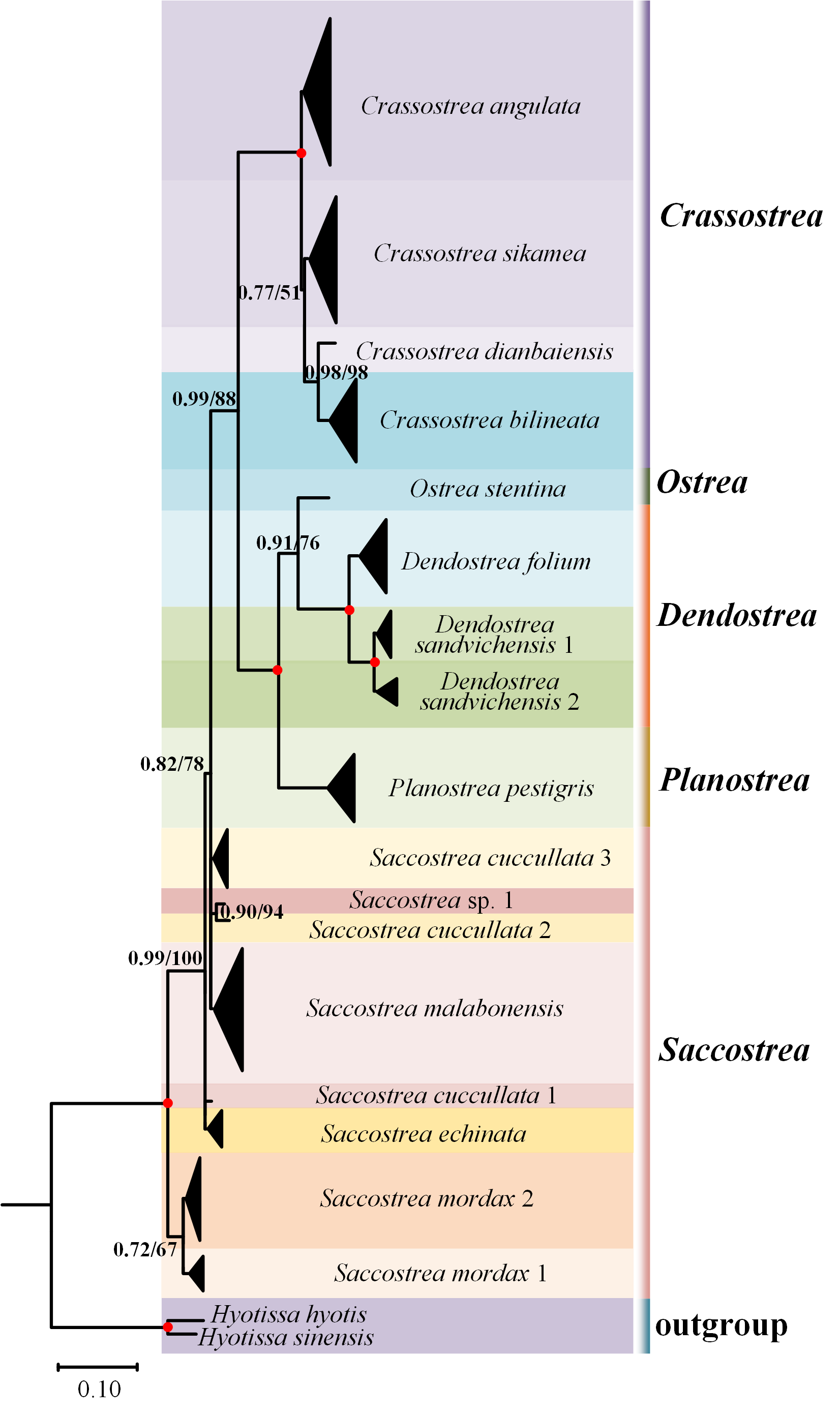

Supplement: Supplementary material 1 — The phylogenetic relationships of the Ostreidae superfamily [file zookeys-1241-247_article-139908__-s001.tif]
