# Supplementary material for: ﻿Species diversity of oysters (Mollusca, Bivalvia) in the intertidal zone of Hainan Island revealed by DNA barcoding analysis
Source: Zookeys. 2025 Jun 13;1241:247–60. doi: 10.3897/zookeys.1241.139908 (PMC12238981; doi:10.3897/zookeys.1241.139908)
Supplement: Supplementary material 3 — The details of Ostreidae specimens used in this study [file zookeys-1241-247_article-139908__-s003.docx]

**Table S1** The details of Ostreidae specimens used in this study.

| Sample number | Species name | Collecting locality | Latitude and longitude | Sampling time | salinity | Accession number | |
| --- | --- | --- | --- | --- | --- | --- | --- |
|  |  |  |  |  |  | COI | 28S |
| 1-1 | *Crassostrea angulata* | Maibang Village, Wenchang(C) | 110°48'28''N 19°28'48''E | February, 2023 | 一 | PQ227257 |  |
| 1-2 | *Crassostrea angulata* | Maibang Village, Wenchang(C) | 110°48'28''N 19°28'48''E | February, 2023 | 一 | PQ227304 |  |
| 1-3 | *Crassostrea angulata* | Maibang Village, Wenchang(C) | 110°48'28''N 19°28'48''E | February, 2023 | 一 | PQ227258 |  |
| 1-6 | *Crassostrea angulata* | Maibang Village, Wenchang(C) | 110°48'28''N 19°28'48''E | February, 2023 | 一 | PQ227259 |  |
| 1-7 | *Crassostrea angulata* | Maibang Village, Wenchang(C) | 110°48'28''N 19°28'48''E | February, 2023 | 一 | PQ227260 |  |
| 1-8 | *Crassostrea angulata* | Maibang Village, Wenchang(C) | 110°48'28''N 19°28'48''E | February, 2023 | 一 | PQ227261 | PQ199320 |
| 1-9 | *Saccostrea malabonensis* | Dongjiao Coconut Forest, Wenchang(B) | 110°50'30''N 19°31'48''E | April, 2023 | 一 | PQ227262 |  |
| 1-11 | *Saccostrea malabonensis* | Dongjiao Coconut Forest, Wenchang(B) | 110°50'30''N 19°31'49''E | April, 2023 | 一 | PQ227263 |  |
| 1-13 | *Saccostrea malabonensis* | Dongjiao Coconut Forest, Wenchang(B) | 110°50'30''N 19°31'50''E | April, 2023 | 一 | PQ227264 |  |
| 1-14 | *Saccostrea malabonensis* | Dongjiao Coconut Forest, Wenchang(B) | 110°50'30''N 19°31'51''E | April, 2023 | 一 | PQ199966 |  |
| 1-16 | *Saccostrea malabonensis* | Dongjiao Coconut Forest, Wenchang(B) | 110°50'30''N 19°31'52''E | April, 2023 | 一 | PQ227265 |  |
| 1-17 | *Saccostrea malabonensis* | Dongjiao Coconut Forest, Wenchang(B) | 110°50'30''N 19°31'53''E | April, 2023 | 一 | PQ227266 |  |
| 1-22 | *Crassostrea sikamea* | Dongjiao Coconut Forest, Wenchang(B) | 110°50'30''N 19°31'54''E | April, 2023 | 一 | PQ199967 | PQ199321 |
| 1-27 | *Crassostrea angulata* | Dongjiao Coconut Forest, Wenchang(B) | 110°50'30''N 19°31'55''E | April, 2023 | 一 | PQ199968 | PQ199322 |
| 1-29 | *Crassostrea angulata* | Xiashan Village, Wenchang(A) | 110°49'48''N 19°58'48''E | April, 2023 | 一 | PQ227267 |  |
| 1-30 | *Planostrea pestigris* | Xiashan Village, Wenchang(A) | 110°49'48''N 19°58'48''E | April, 2023 | 一 | PQ227275 | PQ199323 |
| 1-31 | *Saccostrea malabonensis* | Xiashan Village, Wenchang(A) | 110°49'48''N 19°58'48''E | April, 2023 | 一 | PQ227276 |  |
| 1-32 | *Saccostrea malabonensis* | Xiashan Village, Wenchang(A) | 110°49'48''N 19°58'48''E | April, 2023 | 一 | PQ227277 |  |
| 1-33 | *Crassostrea angulata* | Xiashan Village, Wenchang(A) | 110°49'48''N 19°58'48''E | April, 2023 | 一 | PQ199969 | PQ199324 |
| 1-34 | *Crassostrea angulata* | Xiashan Village, Wenchang(A) | 110°49'48''N 19°58'48''E | April, 2023 | 一 | PQ227268 |  |
| 1-35 | *Crassostrea sikamea* | Xiashan Village, Wenchang(A) | 110°49'48''N 19°58'48''E | April, 2023 | 一 | PQ227270 |  |
| 1-36 | *Saccostrea malabonensis* | Xiashan Village, Wenchang(A) | 110°49'48''N 19°58'48''E | April, 2023 | 一 | PQ227278 |  |
| 1-37 | *Saccostrea malabonensis* | Xiashan Village, Wenchang(A) | 110°49'48''N 19°58'48''E | April, 2023 | 一 | PQ199970 |  |
| 1-38 | *Crassostrea sikamea* | Xiashan Village, Wenchang(A) | 110°49'48''N 19°58'48''E | April, 2023 | 一 | PQ227271 |  |
| 1-39 | *Crassostrea sikamea* | Xiashan Village, Wenchang(A) | 110°49'48''N 19°58'48''E | April, 2023 | 一 | PQ199971 | PQ199325 |
| 1-40 | *Crassostrea angulata* | Xiashan Village, Wenchang(A) | 110°49'48''N 19°58'48''E | April, 2023 | 一 | PQ199972 | PQ199326 |
| 1-41 | *Crassostrea angulata* | Xiashan Village, Wenchang(A) | 110°49'48''N 19°58'48''E | April, 2023 | 一 | PQ199973 |  |
| 1-42 | *Crassostrea sikamea* | Xiashan Village, Wenchang(A) | 110°49'48''N 19°58'48''E | April, 2023 | 一 | PQ227272 |  |
| 1-43 | *Crassostrea angulata* | Xiashan Village, Wenchang(A) | 110°49'48''N 19°58'48''E | April, 2023 | 一 | PQ227269 |  |
| 1-44 | *Crassostrea sikamea* | Xiashan Village, Wenchang(A) | 110°49'48''N 19°58'48''E | April, 2023 | 一 | PQ227273 |  |
| 1-45 | *Saccostrea malabonensis* | Xiashan Village, Wenchang(A) | 110°49'48''N 19°58'48''E | April, 2023 | 一 | PQ227279 | PQ199327 |
| 1-48 | *Crassostrea sikamea* | Xiashan Village, Wenchang(A) | 110°49'48''N 19°58'48''E | April, 2023 | 一 | PQ227274 |  |
| 1-51 | *Saccostrea malabonensis* | Xiashan Village, Wenchang(A) | 110°49'48''N 19°58'48''E | April, 2023 | 一 | PQ227280 |  |
| 1-131 | *Saccostrea malabonensis* | Huiwen Town, Wenchang(Q) | 110°43'12''N  19°27'36''E | April, 2024 | 32 | PQ227281 |  |
| 1-132 | *Saccostrea malabonensis* | Huiwen Town, Wenchang(Q) | 110°43'12''N 19°27'36''E | April, 2024 | 32 | PQ227282 |  |
| 1-133 | *Saccostrea malabonensis* | Huiwen Town, Wenchang(Q) | 110°43'12''N 19°27'36''E | April, 2024 | 32 | PQ227283 |  |
| 1-135 | *Saccostrea malabonensis* | Huiwen Town, Wenchang(Q) | 110°43'12''N 19°27'36''E | April, 2024 | 32 | PQ227284 |  |
| 1-137 | *Saccostrea echinata* | Huiwen Town, Wenchang(Q) | 110°43'12''N 19°27'36''E | April, 2024 | 32 | PQ201000 |  |
| 1-144 | *Saccostrea malabonensis* | Huiwen Town, Wenchang(Q) | 110°43'12''N 19°27'36''E | April, 2024 | 32 | PQ227285 |  |
| 1-145 | *Saccostrea malabonensis* | Huiwen Town, Wenchang(Q) | 110°43'12''N 19°27'36''E | April, 2024 | 32 | PQ227286 |  |
| 1-147 | *Saccostrea echinata* | Huiwen Town, Wenchang(Q) | 110°43'12''N 19°27'36''E | April, 2024 | 32 | PQ201001 |  |
| 2-1 | *Saccostrea cuccullata* 3 | Longjing Beach, Danzhou(S) | 109°16'12''N 19°54'36''E | June, 2023 | 30.1 | PQ227293 |  |
| 2-2 | *Saccostrea cuccullata* 3 | Longjing Beach, Danzhou(S) | 109°16'12''N 19°54'36''E | June, 2023 | 30.1 | PQ227294 |  |
| 2-5 | *Dendostrea sandvichensis* 1 | Longjing Beach, Danzhou(S) | 109°16'12''N 19°54'36''E | June, 2023 | 30.1 | PQ227287 | PQ199328 |
| 2-6 | *Dendostrea sandvichensis* 1 | Longjing Beach, Danzhou(S) | 109°16'12''N 19°54'36''E | June, 2023 | 30.1 | PQ227288 | PQ199329 |
| 2-7 | *Dendostrea sandvichensis* 1 | Longjing Beach, Danzhou(S) | 109°16'12''N 19°54'36''E | June, 2023 | 30.1 | PQ227289 | PQ199384 |
| 2-8 | *Dendostrea sandvichensis* 1 | Longjing Beach, Danzhou(S) | 109°16'12''N 19°54'36''E | June, 2023 | 30.1 | PQ227290 | PQ199385 |
| 2-9 | *Dendostrea sandvichensis* | Longjing Beach, Danzhou(S) | 109°16'12''N 19°54'36''E | June, 2023 | 30.1 | PQ227291 | PQ199386 |
| 2-11 | *Dendostrea sandvichensis* 1 | Longjing Beach, Danzhou(S) | 109°16'12''N 19°54'36''E | June, 2023 | 30.1 | PQ227292 | PQ199387 |
| 2-12 | *Saccostrea mordax* 2 | Longjing Beach, Danzhou(S) | 109°16'12''N 19°54'36''E | June, 2023 | 30.1 | PQ199977 | PQ199330 |
| 2-26 | *Crassostrea bilineata* | Longjing Beach, Danzhou(S) | 109°21'36''N 19°53'12''E | June, 2023 | 29.2 | PQ227299 | PQ199331 |
| 2-27 | *Saccostrea malabonensis* | Longjing Beach, Danzhou(S) | 109°21'36''N 19°53'12''E | June, 2023 | 29.2 | PQ227295 |  |
| 2-30 | *Saccostrea malabonensis* | Longjing Beach, Danzhou(S) | 109°21'36''N 19°53'12''E | June, 2023 | 29.2 | PQ227296 | PQ199332 |
| 2-31 | *Saccostrea echinata* | Longjing Beach, Danzhou(S) | 109°21'36''N 19°53'12''E | June, 2023 | 29.2 | PQ227300 |  |
| 2-32 | *Saccostrea malabonensis* | Longjing Beach, Danzhou(S) | 109°21'36''N 19°53'12''E | June, 2023 | 29.2 | PQ227297 |  |
| 2-35 | *Saccostrea cuccullata* 3 | Xiaoyu Village, Danzhou(T) | 109°21'36''N 19°53'12''E | June, 2023 | 29.2 | PQ227301 |  |
| 2-37 | *Saccostrea malabonensis* | Xiaoyu Village, Danzhou(T) | 109°21'36''N 19°53'12''E | June, 2023 | 29.2 | PQ227298 |  |
| 2-39 | *Saccostrea echinata* | Xiaoyu Village, Danzhou(T) | 109°21'36''N 19°53'12''E | June, 2023 | 29.2 | PQ227302 |  |
| 2-52 | *Saccostrea cuccullata* 3 | Haitou Town, Danzhou(R) | 108°57'36''N 19°31'48''E | June, 2023 | 29.2 | PQ200772 |  |
| 2-55 | *Saccostrea malabonensis* | Haitou Town, Danzhou(R) | 108°57'36''N 19°31'48''E | June, 2023 | 29.2 | PQ227303 | PQ199374 |
| 2-56 | *Saccostrea malabonensis* | Haitou Town, Danzhou(R) | 108°57'36''N 19°31'48''E | June, 2023 | 29.2 | PQ200767 |  |
| 2-57 | *Saccostrea malabonensis* | Haitou Town, Danzhou(R) | 108°57'36''N 19°31'48''E | June, 2023 | 29.2 | PQ200768 |  |
| 2-58 | *Saccostrea malabonensis* | Haitou Town, Danzhou(R) | 108°57'36''N 19°31'48''E | June, 2023 | 29.2 | PQ200769 |  |
| 2-61 | *Saccostrea malabonensis* | Haitou Town, Danzhou(R) | 108°57'36''N 19°31'48''E | June, 2023 | 29.2 | PQ200770 |  |
| 2-64 | *Saccostrea cuccullata* 3 | Haitou Town, Danzhou(R) | 108°57'36''N 19°31'48''E | June, 2023 | 29.2 | PQ200773 |  |
| 2-65 | *Saccostrea malabonensis* | Haitou Town, Danzhou(R) | 108°57'36''N 19°31'48''E | June, 2023 | 29.2 | PQ200771 |  |
| 2-66 | *Saccostrea cucullate* 2 | Haitou Town, Danzhou(R) | 108°57'36''N 19°31'48''E | June, 2023 | 29.2 | PQ200774 |  |
| 2-75 | *Saccostrea cucullate* 2 | Haitou Town, Danzhou(R) | 108°57'36''N 19°31'48''E | June, 2023 | 29.2 | PQ199978 |  |
| 2-80 | *Saccostrea cuccullata* 3 | Haitou Town, Danzhou(R) | 108°57'36''N 19°31'48''E | May, 2024 | 30.0 | PQ201002 |  |
| 2-81 | *Saccostrea cuccullata* 3 | Haitou Town, Danzhou(R) | 108°57'36''N 19°31'48''E | May, 2024 | 30.0 | PQ201003 |  |
| 2-82 | *Saccostrea cuccullata* 3 | Haitou Town, Danzhou(R) | 108°57'36''N 19°31'48''E | May, 2024 | 30.0 | PQ201004 |  |
| 2-84 | *Saccostrea cuccullata* 3 | Haitou Town, Danzhou(R) | 108°57'36''N 19°31'48''E | May, 2024 | 30.0 | PQ201005 |  |
| 2-85 | *Saccostrea cuccullata* 3 | Haitou Town, Danzhou(R) | 108°57'36''N 19°31'48''E | May, 2024 | 30.0 | PQ201006 |  |
| 2-86 | *Saccostrea cuccullata* 3 | Haitou Town, Danzhou(R) | 108°57'36''N 19°31'48''E | May, 2024 | 30.0 | PQ201007 |  |
| 2-87 | *Saccostrea malabonensis* | Haitou Town, Danzhou(R) | 108°57'36''N 19°31'48''E | May, 2024 | 30.0 | PQ200777 |  |
| 2-90 | *Saccostrea malabonensis* | Haitou Town, Danzhou(R) | 108°57'36''N 19°31'48''E | May, 2024 | 30.0 | PQ200778 |  |
| 2-92 | *Saccostrea malabonensis* | Haitou Town, Danzhou(R) | 108°57'36''N 19°31'48''E | May, 2024 | 30.0 | PQ200779 |  |
| 2-93 | *Saccostrea malabonensis* | Haitou Town, Danzhou(R) | 108°57'36''N 19°31'48''E | May, 2024 | 30.0 | PQ200775 |  |
| 2-97 | *Saccostrea malabonensis* | Haitou Town, Danzhou(R) | 108°57'36''N 19°31'48''E | May, 2024 | 30.0 | PQ200776 |  |
| 3-2 | *Saccostrea mordax* 2 | Zhongjiao Park, Changjiang(O) | 108°40'12''N 19°21'36''E | June, 2023 | 29.2 | PQ199979 |  |
| 3-4 | *Saccostrea mordax* 2 | Zhongjiao Park, Changjiang(O) | 108°40'12''N 19°21'36''E | June, 2023 | 29.2 | PQ200780 |  |
| 3-5 | *Saccostrea mordax* 2 | Zhongjiao Park, Changjiang(O) | 108°40'12''N 19°21'36''E | June, 2023 | 29.2 | PQ200781 | PQ199333 |
| 3-7 | *Saccostrea mordax* 2 | Zhongjiao Park, Changjiang(O) | 108°40'12''N 19°21'36''E | June, 2023 | 29.2 | PQ200782 | PQ199334 |
| 3-8 | *Saccostrea malabonensis* | Haiwei Town,Changjiang(Q) | 108°53'24''N 19°28'12''E | May, 2023 | 26.6 | PQ200783 |  |
| 3-9 | *Crassostrea sikamea* | Haiwei Town,Changjiang(Q) | 108°53'24''N 19°28'12''E | May, 2023 | 26.6 | PQ200784 | PQ199335 |
| 3-10 | *Crassostrea sikamea* | Haiwei Town,Changjiang(Q) | 108°53'24''N 19°28'12''E | May, 2023 | 26.6 | PQ200785 | PQ199336 |
| 3-12 | *Saccostrea malabonensis* | Haiwei Town,Changjiang(Q) | 108°53'24''N 19°28'12''E | May, 2023 | 26.6 | PQ200786 |  |
| 3-13 | *Saccostrea malabonensis* | Haiwei Town,Changjiang(Q) | 108°53'24''N 19°28'12''E | May, 2023 | 26.6 | PQ200787 | PQ199337 |
| 3-15 | *Saccostrea cuccullata* 3 | Haiwei Town,Changjiang(Q) | 108°53'24''N 19°28'12''E | May, 2023 | 26.6 | PQ200790 | PQ199383 |
| 3-16 | *Crassostrea bilineata* | Haiwei Town,Changjiang(Q) | 108°53'24''N 19°28'12''E | May, 2023 | 26.6 | PQ200789 |  |
| 3-17 | *Saccostrea malabonensis* | Haiwei Town,Changjiang(Q) | 108°53'24''N 19°28'12''E | May, 2023 | 26.6 | PQ200788 |  |
| 3-29 | *Saccostrea echinata* | Haiwei Town,Changjiang(Q) | 108°53'24''N 19°28'12''E | September, 2023 | 30.3 | PQ200791 |  |
| 3-31 | *Saccostrea mordax* 2 | Haiwei Town,Changjiang(Q) | 108°53'24''N 19°28'12''E | September, 2023 | 30.3 | PQ200792 | PQ199372 |
| 3-32 | *Saccostrea mordax* 2 | Haiwei Town,Changjiang(Q) | 108°53'24''N 19°28'12''E | September, 2023 | 30.3 | PQ200793 |  |
| 3-34 | *Saccostrea echinata* | Haiwei Town,Changjiang(Q) | 108°53'24''N 19°28'12''E | September, 2023 | 30.3 | PQ200796 |  |
| 3-35 | *Saccostrea mordax* 2 | Haiwei Town,Changjiang(Q) | 108°53'24''N 19°28'12''E | September, 2023 | 30.3 | PQ200794 |  |
| 3-36 | *Saccostrea mordax* 2 | Haiwei Town,Changjiang(Q) | 108°53'24''N 19°28'12''E | September, 2023 | 30.3 | PQ200795 |  |
| 3-40 | *Saccostrea mordax* 2 | Haiwei Town,Changjiang(Q) | 108°53'24''N 19°28'12''E | September, 2023 | 30.3 | PQ200797 |  |
| 3-41 | *Saccostrea mordax* 2 | Haiwei Town,Changjiang(Q) | 108°53'24''N 19°28'13''E | September, 2023 | 30.3 | PQ200798 |  |
| 3-44 | *Saccostrea malabonensis* | Guohe Yuan,Changjiang(P) | 108°38'24''N 19°28'12''E | January, 2024 | 14.0 | PQ200799 |  |
| 3-47 | *Saccostrea malabonensis* | Haiwei Town,Changjiang(Q) | 108°53'24''N 19°28'12''E | May, 2024 | 34.0 | PQ200800 |  |
| 3-53 | *Saccostrea cuccullata* 3 | Haiwei Town,Changjiang(Q) | 108°53'24''N 19°28'12''E | May, 2024 | 34.0 | PQ201011 |  |
| 3-54 | *Saccostrea malabonensis* | Haiwei Town,Changjiang(Q) | 108°53'24''N 19°28'12''E | May, 2024 | 34.0 | PQ200801 |  |
| 3-57 | *Saccostrea cuccullata* 3 | Haiwei Town,Changjiang(Q) | 108°53'24''N 19°28'12''E | May, 2024 | 34.0 | PQ201008 |  |
| 3-58 | *Saccostrea cuccullata* 3 | Haiwei Town,Changjiang(Q) | 108°53'24''N 19°28'12''E | May, 2024 | 34.0 | PQ200816 | PQ199388 |
| 3-59 | *Saccostrea malabonensis* | Haiwei Town,Changjiang(Q) | 108°53'24''N 19°28'12''E | May, 2024 | 34.0 | PQ200802 |  |
| 3-60 | *Saccostrea cuccullata* 3 | Haiwei Town,Changjiang(Q) | 108°53'24''N 19°28'12''E | May, 2024 | 34.0 | PQ199980 |  |
| 3-61 | *Saccostrea cuccullata* 3 | Haiwei Town,Changjiang(Q) | 108°53'24''N 19°28'12''E | May, 2024 | 34.0 | PQ201009 |  |
| 3-63 | *Saccostrea malabonensis* | Haiwei Town,Changjiang(Q) | 108°53'24''N 19°28'12''E | May, 2024 | 34.0 | PQ200803 |  |
| 3-65 | *Saccostrea malabonensis* | Haiwei Town,Changjiang(Q) | 108°53'24''N 19°28'12''E | May, 2024 | 34.0 | PQ200804 |  |
| 3-66 | *Saccostrea malabonensis* | Haiwei Town,Changjiang(Q) | 108°53'24''N 19°28'12''E | May, 2024 | 34.0 | PQ200805 |  |
| 3-97 | *Crassostrea bilineata* | Guohe Yuan,Changjiang(P) | 108°38'24''N 19°28'12''E | May, 2024 | 14.0 | PQ200810 |  |
| 3-98 | *Crassostrea bilineata* | Guohe Yuan,Changjiang(P) | 108°38'24''N 19°28'12''E | May, 2024 | 14.0 | PQ200811 |  |
| 3-99 | *Saccostrea malabonensis* | Guohe Yuan,Changjiang(P) | 108°38'24''N 19°28'12''E | May, 2024 | 14.0 | PQ200806 |  |
| 3-100 | *Crassostrea bilineata* | Guohe Yuan,Changjiang(P) | 108°38'24''N 19°28'12''E | May, 2024 | 14.0 | PQ200812 |  |
| 3-101 | *Crassostrea bilineata* | Guohe Yuan,Changjiang(P) | 108°38'24''N 19°28'12''E | May, 2024 | 14.0 | PQ200813 |  |
| 3-102 | *Saccostrea malabonensis* | Guohe Yuan,Changjiang(P) | 108°38'24''N 19°28'12''E | May, 2024 | 14.0 | PQ200807 |  |
| 3-105 | *Saccostrea malabonensis* | Guohe Yuan,Changjiang(P) | 108°38'24''N 19°28'12''E | May, 2024 | 14.0 | PQ200808 |  |
| 3-106 | *Saccostrea malabonensis* | Guohe Yuan,Changjiang(P) | 108°38'24''N 19°28'12''E | May, 2024 | 14.0 | PQ200809 |  |
| 3-111 | *Crassostrea bilineata* | Guohe Yuan,Changjiang(P) | 108°38'24''N 19°28'12''E | May, 2024 | 14.0 | PQ200814 |  |
| 3-114 | *Crassostrea sikamea* | Guohe Yuan,Changjiang(P) | 108°38'24''N 19°28'12''E | May, 2024 | 14.0 | PQ200815 |  |
| 3-115 | *Saccostrea cuccullata* 3 | Guohe Yuan,Changjiang(P) | 108°38'24''N 19°28'12''E | May, 2024 | 14.0 | PQ201010 |  |
| 4-2 | *Crassostrea angulata* | Longhao Village, Lingao(V) | 109°42'36''N  20°0'36''E | January, 2023 | 一 | PQ200817 | PQ199338 |
| 4-4 | *Crassostrea angulata* | Longhao Village, Lingao(V) | 109°42'36''N  20°0'36''E | January, 2023 | 一 | PQ200818 | PQ199339 |
| 4-5 | *Crassostrea angulata* | Longhao Village, Lingao(V) | 109°42'36''N  20°0'36''E | January, 2023 | 一 | PQ200819 |  |
| 4-6 | *Crassostrea sikamea* | Longhao Village, Lingao(V) | 109°42'36''N  20°0'36''E | January, 2023 | 一 | PQ200820 | PQ199340 |
| 4-7 | *Saccostrea malabonensis* | Wenlanjiang Bridge, Lingao(W) | 109°44'24''N 19°58'48''E | June, 2023 | 13.0 | PQ200823 | PQ199341 |
| 4-8 | *Saccostrea malabonensis* | Wenlanjiang Bridge, Lingao(W) | 109°44'24''N 19°58'48''E | June, 2023 | 13.0 | PQ200824 |  |
| 4-9 | *Crassostrea bilineata* | Wenlanjiang Bridge, Lingao(W) | 109°44'24''N 19°58'48''E | June, 2023 | 13.0 | PQ200826 | PQ199342 |
| 4-10 | *Crassostrea bilineata* | Wenlanjiang Bridge, Lingao(W) | 109°44'24''N 19°58'48''E | June, 2023 | 13.0 | PQ200827 |  |
| 4-11 | *Crassostrea angulata* | Wenlanjiang Bridge, Lingao(W) | 109°44'24''N 19°58'48''E | June, 2023 | 13.0 | PQ200821 | PQ199343 |
| 4-12 | *Crassostrea bilineata* | Wenlanjiang Bridge, Lingao(W) | 109°44'24''N 19°58'48''E | June, 2023 | 13.0 | PQ200828 |  |
| 4-13 | *Crassostrea bilineata* | Wenlanjiang Bridge, Lingao(W) | 109°44'24''N 19°58'48''E | June, 2023 | 13.0 | PQ200829 | PQ199344 |
| 4-14 | *Crassostrea bilineata* | Wenlanjiang Bridge, Lingao(W) | 109°44'24''N 19°58'48''E | June, 2023 | 13.0 | PQ200830 |  |
| 4-15 | *Crassostrea angulata* | Wenlanjiang Bridge, Lingao(W) | 109°44'24''N 19°58'48''E | June, 2023 | 13.0 | PQ200822 | PQ199345 |
| 4-16 | *Saccostrea malabonensis* | Wenlanjiang Bridge, Lingao(W) | 109°44'24''N 19°58'48''E | June, 2023 | 13.0 | PQ200825 |  |
| 4-17 | *Crassostrea bilineata* | Wenlanjiang Bridge, Lingao(W) | 109°44'24''N 19°58'48''E | June, 2023 | 13.0 | PQ200831 |  |
| 4-34 | *Saccostrea cuccullata* 3 | Bozhong Village, Lingao(U) | 109°35'24''N 19°58'48''E | June, 2023 | 30.2 | PQ200832 |  |
| 4-35 | *Dendostrea sandvichensis* 1 | Bozhong Village, Lingao(U) | 109°35'24''N 19°58'48''E | June, 2023 | 30.2 | PQ200841 | PQ199346 |
| 4-36 | *Dendostrea folium* | Bozhong Village, Lingao(U) | 109°35'24''N 19°58'48''E | June, 2023 | 30.2 | PQ200835 |  |
| 4-39 | *Dendostrea sandvichensis* 2 | Bozhong Village, Lingao(U) | 109°35'24''N 19°58'48''E | June, 2023 | 30.2 | PQ200842 | PQ199347 |
| 4-40 | *Dendostrea folium* | Bozhong Village, Lingao(U) | 109°35'24''N 19°58'48''E | June, 2023 | 30.2 | PQ200836 | PQ199348 |
| 4-41 | *Saccostrea malabonensis* | Bozhong Village, Lingao(U) | 109°35'24''N 19°58'48''E | June, 2023 | 30.2 | PQ201012 |  |
| 4-42 | *Dendostrea folium* | Bozhong Village, Lingao(U) | 109°35'24''N 19°58'48''E | June, 2023 | 30.2 | PQ200837 |  |
| 4-48 | *Saccostrea malabonensis* | Bozhong Village, Lingao(U) | 109°35'24''N 19°58'48''E | June, 2023 | 30.2 | PQ200833 | PQ199349 |
| 4-49 | *Dendostrea folium* | Bozhong Village, Lingao(U) | 109°35'24''N 19°58'48''E | June, 2023 | 30.2 | PQ200838 |  |
| 4-50 | *Saccostrea echinata* | Bozhong Village, Lingao(U) | 109°35'24''N 19°58'48''E | June, 2023 | 30.2 | PQ200840 | PQ199379 |
| 4-53 | *Dendostrea folium* | Bozhong Village, Lingao(U) | 109°35'24''N 19°58'48''E | June, 2023 | 30.2 | PQ200839 |  |
| 4-55 | *Saccostrea malabonensis* | Bozhong Village, Lingao(U) | 109°35'24''N 19°58'48''E | June, 2023 | 30.2 | PQ200834 | PQ199381 |
| 4-56 | *Dendostrea folium* | Bozhong Village, Lingao(U) | 109°35'24''N 19°58'48''E | June, 2023 | 30.2 | PQ200844 | PQ199389 |
| 4-60 | *Ostrea stentina* | Bozhong Village, Lingao(U) | 109°35'24''N 19°58'48''E | June, 2023 | 30.2 | PQ200843 | PQ199350 |
| 4-91 | *Saccostrea malabonensis* | Wenlanjiang Bridge, Lingao(W) | 109°44'24''N 19°58'48''E | May, 2024 | 12.0 | PQ200845 |  |
| 4-92 | *Saccostrea malabonensis* | Wenlanjiang Bridge, Lingao(W) | 109°44'24''N 19°58'48''E | May, 2024 | 12.0 | PQ200846 |  |
| 4-93 | *Saccostrea malabonensis* | Wenlanjiang Bridge, Lingao(W) | 109°44'24''N 19°58'48''E | May, 2024 | 12.0 | PQ200847 |  |
| 4-107 | *Crassostrea bilineata* | Wenlanjiang Bridge, Lingao(W) | 109°44'24''N 19°58'48''E | May, 2024 | 12.0 | PQ200848 |  |
| 4-109 | *Crassostrea bilineata* | Wenlanjiang Bridge, Lingao(W) | 109°44'24''N 19°58'48''E | May, 2024 | 12.0 | PQ226030 |  |
| 5-1 | *Saccostrea mordax* 2 | Wuzhizhou Island,Sanya(I) | 109°46'48''N 18°18'36''E | August, 2023 | 28.9 | PQ226031 | PQ199373 |
| 5-6 | *Saccostrea mordax* 2 | Wuzhizhou Island,Sanya(I) | 109°46'48''N 18°18'36''E | August, 2023 | 28.9 | PQ226050 | PQ199375 |
| 5-9 | *Saccostrea mordax* 1 | Wuzhizhou Island,Sanya(I) | 109°46'48''N 18°18'36''E | August, 2023 | 28.9 | PQ226059 | PQ199396 |
| 5-13 | *Saccostrea mordax* 2 | Wuzhizhou Island,Sanya(I) | 109°46'48''N 18°18'36''E | Aug-23 | 28.9 | PQ226034 |  |
| 5-14 | *Saccostrea mordax* 1 | Wuzhizhou Island,Sanya(I) | 109°46'48''N 18°18'36''E | Aug-23 | 28.9 | PQ226039 |  |
| 5-16 | *Saccostrea mordax* 1 | Wuzhizhou Island,Sanya(I) | 109°46'48''N 18°18'36''E | Aug-23 | 28.9 | PQ226044 | PQ199390 |
| 5-17 | *Saccostrea mordax* 1 | Wuzhizhou Island,Sanya(I) | 109°46'48''N 18°18'36''E | Aug-23 | 28.9 | PQ226045 | PQ199391 |
| 5-44 | *Saccostrea mordax* 2 | Dadonghai,Sanya(J) | 109°31'48''N 18°13'12''E | Aug-23 | 29.8 | PQ226046 |  |
| 5-45 | *Saccostrea mordax* 2 | Dadonghai,Sanya(J) | 109°31'48''N 18°13'12''E | Aug-23 | 29.8 | PQ226047 |  |
| 5-46 | *Saccostrea mordax* 1 | Dadonghai,Sanya(J) | 109°31'48''N 18°13'12''E | Aug-23 | 29.8 | PQ226048 | PQ199376 |
| 5-47 | *Saccostrea mordax* 2 | Dadonghai,Sanya(J) | 109°31'48''N 18°13'12''E | Aug-23 | 29.8 | PQ226049 |  |
| 5-71 | *Saccostrea malabonensis* | Luhuitou Scenic Area,Sanya(L) | 109°30'36''N 18°12'36''E | September, 2023 | 29.8 | PQ226051 |  |
| 5-72 | *Saccostrea malabonensis* | Luhuitou Scenic Area,Sanya(L) | 109°30'36''N 18°12'36''E | September, 2023 | 29.8 | PQ226052 |  |
| 5-75 | *Saccostrea malabonensis* | Luhuitou Scenic Area,Sanya(L) | 109°30'36''N 18°12'36''E | September, 2023 | 29.8 | PQ226053 |  |
| 5-76 | *Saccostrea mordax* 2 | Luhuitou Scenic Area,Sanya(L) | 109°30'36''N 18°12'36''E | September, 2023 | 29.8 | PQ226054 |  |
| 5-77 | *Saccostrea mordax* 1 | Luhuitou Scenic Area,Sanya(L) | 109°30'36''N 18°12'36''E | September, 2023 | 29.8 | PQ226055 | PQ199377 |
| 5-78 | *Saccostrea mordax* 1 | Luhuitou Scenic Area,Sanya(L) | 109°30'36''N 18°12'36''E | September, 2023 | 29.8 | PQ226056 | PQ199397 |
| 5-79 | *Saccostrea mordax* 2 | Luhuitou Scenic Area,Sanya(L) | 109°30'36''N 18°12'36''E | September, 2023 | 29.8 | PQ226057 |  |
| 5-80 | *Saccostrea mordax* 2 | Luhuitou Scenic Area,Sanya(L) | 109°30'36''N 18°12'36''E | September, 2023 | 29.8 | PQ226058 |  |
| 5-128 | *Crassostrea sikamea* | Hongzhou Pier,Sanya(K) | 109°30'36''N 18°13'48''E | January, 2024 | 29.0 | PQ226032 |  |
| 5-129 | *Crassostrea bilineata* | Hongzhou Pier,Sanya(K) | 109°30'36''N 18°13'48''E | January, 2024 | 29.0 | PQ226033 |  |
| 5-130 | *Crassostrea bilineata* | Hongzhou Pier,Sanya(K) | 109°30'36''N 18°13'48''E | January, 2024 | 29.0 | PQ226035 |  |
| 5-137 | *Saccostrea malabonensis* | Hongzhou Pier,Sanya(K) | 109°30'36''N 18°13'48''E | April, 2024 | 25.0 | PQ226036 |  |
| 5-138 | *Saccostrea malabonensis* | Hongzhou Pier,Sanya(K) | 109°30'36''N 18°13'48''E | April, 2024 | 25.0 | PQ226037 |  |
| 5-139 | *Saccostrea malabonensis* | Hongzhou Pier,Sanya(K) | 109°30'36''N 18°13'48''E | April, 2024 | 25.0 | PQ226038 |  |
| 5-140 | *Crassostrea bilineata* | Hongzhou Pier,Sanya(K) | 109°30'36''N 18°13'48''E | April, 2024 | 25.0 | PQ226040 |  |
| 5-141 | *Saccostrea malabonensis* | Hongzhou Pier,Sanya(K) | 109°30'36''N 18°13'48''E | April, 2024 | 25.0 | PQ226041 |  |
| 5-142 | *Saccostrea malabonensis* | Hongzhou Pier,Sanya(K) | 109°30'36''N 18°13'48''E | April, 2024 | 25.0 | PQ226042 |  |
| 5-143 | *Saccostrea malabonensis* | Hongzhou Pier,Sanya(K) | 109°30'36''N 18°13'48''E | April, 2024 | 25.0 | PQ226043 |  |
| 6-1 | *Crassostrea angulata* | Shiji Bridge, Haikou(Y) | 110°19'12''N 20°3'36''E | March, 2023 | 一 | PQ226060 | PQ199351 |
| 6-2 | *Crassostrea sikamea* | Shiji Bridge, Haikou(Y) | 110°19'12''N 20°3'36''E | March, 2023 | 一 | PQ226067 | PQ199352 |
| 6-3 | *Crassostrea angulata* | Shiji Bridge, Haikou(Y) | 110°19'12''N 20°3'36''E | March, 2023 | 一 | PQ226068 |  |
| 6-6 | *Saccostrea malabonensis* | Baishamen Park, Haikou(Z) | 110°20'24''N 20°4'48''E | March, 2024 | 30.0 | PQ226074 |  |
| 6-7 | *Crassostrea sikamea* | Baishamen Park, Haikou(Z) | 110°20'24''N 20°4'48''E | March, 2024 | 30.0 | PQ226075 |  |
| 6-8 | *Crassostrea angulata* | Baishamen Park, Haikou(Z) | 110°20'24''N 20°4'48''E | March, 2024 | 30.0 | PQ226076 |  |
| 6-9 | *Crassostrea angulata* | Baishamen Park, Haikou(Z) | 110°20'24''N 20°4'48''E | March, 2024 | 30.0 | PQ226077 |  |
| 6-10 | *Crassostrea angulata* | Baishamen Park, Haikou(Z) | 110°20'24''N 20°4'48''E | March, 2024 | 30.0 | PQ226061 |  |
| 6-11 | *Crassostrea sikamea* | Baishamen Park, Haikou(Z) | 110°20'24''N 20°4'48''E | March, 2024 | 30.0 | PQ226062 |  |
| 6-12 | *Saccostrea echinata* | Baishamen Park, Haikou(Z) | 110°20'24''N 20°4'48''E | March, 2024 | 30.0 | PQ226063 |  |
| 6-13 | *Crassostrea angulata* | Baishamen Park, Haikou(Z) | 110°20'24''N 20°4'48''E | March, 2024 | 30.0 | PQ226064 |  |
| 6-14 | *Crassostrea angulata* | Baishamen Park, Haikou(Z) | 110°20'24''N 20°4'48''E | March, 2024 | 30.0 | PQ226065 |  |
| 6-15 | *Crassostrea angulata* | Baishamen Park, Haikou(Z) | 110°20'24''N 20°4'48''E | March, 2024 | 30.0 | PQ226066 |  |
| 6-31 | *Crassostrea sikamea* | Baishamen Park, Haikou(Z) | 110°20'24''N 20°4'48''E | March, 2024 | 30.0 | PQ226069 |  |
| 6-32 | *Crassostrea angulata* | Baishamen Park, Haikou(Z) | 110°20'24''N 20°4'48''E | March, 2024 | 30.0 | PQ226070 |  |
| 6-33 | *Crassostrea sikamea* | Baishamen Park, Haikou(Z) | 110°20'24''N 20°4'48''E | March, 2024 | 30.0 | PQ226071 |  |
| 6-35 | *Saccostrea echinata* | Baishamen Park, Haikou(Z) | 110°20'24''N 20°4'48''E | March, 2024 | 30.0 | PQ226072 |  |
| 6-36 | *Crassostrea sikamea* | Baishamen Park, Haikou(Z) | 110°20'24''N 20°4'48''E | March, 2024 | 30.0 | PQ226073 |  |
| 7-1 | *Saccostrea malabonensis* | Wanquan River Estuary , Qionghai(F) | 110°35'24''N 19°8'24''E | April, 2023 | 一 | PQ199981 |  |
| 7-2 | *Saccostrea malabonensis* | Wanquan River Estuary , Qionghai(F) | 110°35'24''N 19°8'24''E | April, 2023 | 一 | PQ226079 |  |
| 7-3 | *Saccostrea malabonensis* | Wanquan River Estuary , Qionghai(F) | 110°35'24''N 19°8'24''E | April, 2023 | 一 | PQ199982 |  |
| 7-4 | *Saccostrea malabonensis* | Wanquan River Estuary , Qionghai(F) | 110°35'24''N 19°8'24''E | April, 2023 | 一 | PQ226086 |  |
| 7-5 | *Saccostrea malabonensis* | Wanquan River Estuary , Qionghai(F) | 110°35'24''N 19°8'24''E | April, 2023 | 一 | PQ199983 |  |
| 7-6 | *Saccostrea malabonensis* | Wanquan River Estuary , Qionghai(F) | 110°35'24''N 19°8'24''E | April, 2023 | 一 | PQ199984 |  |
| 7-7 | *Crassostrea dianbaiensis* | Wanquan River Estuary , Qionghai(F) | 110°35'24''N 19°8'24''E | April, 2023 | 一 | PQ226087 | PQ199353 |
| 7-14 | *Crassostrea bilineata* | Wanquan River Estuary , Qionghai(F) | 110°35'24''N 19°8'24''E | April, 2023 | 一 | PQ226078 | PQ199354 |
| 7-21 | *Saccostrea malabonensis* | Changpo Village, Qionghai(E) | 110°41'24''N 19°21'36''E | April, 2024 | 31 | PQ226080 |  |
| 7-22 | *Saccostrea malabonensis* | Changpo Village, Qionghai(E) | 110°41'24''N 19°21'36''E | April, 2024 | 31 | PQ226081 |  |
| 7-23 | *Saccostrea malabonensis* | Changpo Village, Qionghai(E) | 110°41'24''N 19°21'36''E | April, 2024 | 31 | PQ226082 |  |
| 7-25 | *Saccostrea malabonensis* | Changpo Village, Qionghai(E) | 110°41'24''N 19°21'36''E | April, 2024 | 31 | PQ226083 |  |
| 7-26 | *Saccostrea malabonensis* | Changpo Village, Qionghai(E) | 110°41'24''N 19°21'36''E | April, 2024 | 31 | PQ226084 |  |
| 7-29 | *Saccostrea malabonensis* | Changpo Village, Qionghai(E) | 110°41'24''N 19°21'36''E | April, 2024 | 31 | PQ226085 |  |
| 10-1 | *Saccostrea* sp. 1 | Gangmenling Park, Lingshui(G) | 110°4'12''N 18°24'36''E | June, 2023 | 30.4 | PQ226011 | PQ199378 |
| 10-2 | *Saccostrea malabonensis* | Gangmenling Park, Lingshui(G) | 110°4'12''N 18°24'36''E | June, 2023 | 30.4 | PQ226021 |  |
| 10-3 | *Saccostrea malabonensis* | Gangmenling Park, Lingshui(G) | 110°4'12''N 18°24'36''E | June, 2023 | 30.4 | PQ226023 |  |
| 10-5 | *Saccostrea mordax* 2 | Gangmenling Park, Lingshui(G) | 110°4'12''N 18°24'36''E | June, 2023 | 30.4 | PQ226025 |  |
| 10-6 | *Saccostrea malabonensis* | Gangmenling Park, Lingshui(G) | 110°4'12''N 18°24'36''E | June, 2023 | 30.4 | PQ226026 |  |
| 10-7 | *Saccostrea mordax* 2 | Gangmenling Park, Lingshui(G) | 110°4'12''N 18°24'36''E | June, 2023 | 30.4 | PQ226027 | PQ199355 |
| 10-8 | *Saccostrea mordax* 2 | Gangmenling Park, Lingshui(G) | 110°4'12''N 18°24'36''E | June, 2023 | 30.4 | PQ226028 | PQ199356 |
| 10-9 | *Saccostrea malabonensis* | Tonghai Village, Lingshui(H) | 110°1'12''N 18°25'48''E | June, 2023 | 30.4 | PQ226029 |  |
| 10-10 | *Saccostrea malabonensis* | Tonghai Village, Lingshui(H) | 110°1'12''N 18°25'48''E | June, 2023 | 29.7 | PQ226012 |  |
| 10-11 | *Saccostrea malabonensis* | Tonghai Village, Lingshui(H) | 110°1'12''N 18°25'48''E | June, 2023 | 29.7 | PQ226013 |  |
| 10-12 | *Planostrea pestigris* | Tonghai Village, Lingshui(H) | 110°1'12''N 18°25'48''E | June, 2023 | 29.7 | PQ226014 | PQ199392 |
| 10-13 | *Planostrea pestigris* | Tonghai Village, Lingshui(H) | 110°1'12''N 18°25'48''E | June, 2023 | 29.7 | PQ226015 | PQ199357 |
| 10-14 | *Planostrea pestigris* | Tonghai Village, Lingshui(H) | 110°1'12''N 18°25'48''E | June, 2023 | 29.7 | PQ226016 | PQ199358 |
| 10-15 | *Crassostrea bilineata* | Tonghai Village, Lingshui(H) | 110°1'12''N 18°25'48''E | June, 2023 | 29.7 | PQ200944 | PQ199359 |
| 10-16 | *Saccostrea malabonensis* | Tonghai Village, Lingshui(H) | 110°1'12''N 18°25'48''E | June, 2023 | 29.7 | PQ226017 |  |
| 10-17 | *Saccostrea malabonensis* | Tonghai Village, Lingshui(H) | 110°1'12''N 18°25'48''E | June, 2023 | 29.7 | PQ226018 |  |
| 10-18 | *Saccostrea malabonensis* | Tonghai Village, Lingshui(H) | 110°1'12''N 18°25'48''E | June, 2023 | 29.7 | PQ226019 | PQ199360 |
| 10-19 | *Saccostrea malabonensis* | Tonghai Village, Lingshui(H) | 110°1'12''N 18°25'48''E | June, 2023 | 29.7 | PQ226020 |  |
| 10-24 | *Crassostrea bilineata* | Tonghai Village, Lingshui(H) | 110°1'12''N 18°25'48''E | June, 2023 | 29.7 | PQ200945 | PQ199361 |
| 10-26 | *Planostrea pestigris* | Tonghai Village, Lingshui(H) | 110°1'12''N 18°25'48''E | June, 2023 | 29.7 | PQ200943 | PQ199393 |
| 10-28 | *Saccostrea malabonensis* | Gangmenling Park, Lingshui(G) | 110°4'21''N 18°24'36''E | June, 2023 | 29.7 | PQ226022 |  |
| 10-30 | *Saccostrea malabonensis* | Gangmenling Park, Lingshui(G) | 110°4'21''N 18°24'36''E | June, 2023 | 30.4 | PQ226024 |  |
| 10-40 | *Saccostrea malabonensis* | Tonghai Village, Lingshui(H) | 110°1'12''N 18°25'48''E | April, 2024 | 29.0 | PQ200946 |  |
| 10-41 | *Saccostrea malabonensis* | Tonghai Village, Lingshui(H) | 110°1'12''N 18°25'48''E | April, 2024 | 29.0 | PQ200947 |  |
| 10-42 | *Saccostrea malabonensis* | Tonghai Village, Lingshui(H) | 110°1'12''N 18°25'48''E | April, 2024 | 29.0 | PQ200948 |  |
| 10-43 | *Crassostrea bilineata* | Tonghai Village, Lingshui(H) | 110°1'12''N 18°25'48''E | April, 2024 | 29.0 | PQ200951 |  |
| 10-44 | *Saccostrea malabonensis* | Tonghai Village, Lingshui(H) | 110°1'12''N 18°25'48''E | April, 2024 | 29.0 | PQ200949 |  |
| 10-45 | *Saccostrea malabonensis* | Tonghai Village, Lingshui(H) | 110°1'12''N 18°25'48''E | April, 2024 | 29.0 | PQ200950 |  |
| 10-46 | *Saccostrea malabonensis* | Tonghai Village, Lingshui(H) | 110°1'12''N 18°25'48''E | April, 2024 | 29.0 | PQ200952 |  |
| 11-1 | *Saccostrea echinata* | Haibin Village, Ledong(M) | 108°42'36''N 18°40'48''E | June, 2023 | 30.8 | PQ200957 | PQ199362 |
| 11-2 | *Saccostrea malabonensis* | Haibin Village, Ledong(M) | 108°42'36''N 18°40'48''E | June, 2023 | 30.8 | PQ200953 |  |
| 11-3 | *Saccostrea echinata* | Haibin Village, Ledong(M) | 108°42'36''N 18°40'48''E | June, 2023 | 30.8 | PQ200958 |  |
| 11-4 | *Crassostrea sikamea* | Haibin Village, Ledong(M) | 108°42'36''N 18°40'48''E | June, 2023 | 30.8 | PQ200961 | PQ199363 |
| 11-5 | *Saccostrea malabonensis* | Haibin Village, Ledong(M) | 108°42'36''N 18°40'48''E | June, 2023 | 30.8 | PQ200954 |  |
| 11-7 | *Crassostrea bilineata* | Haibin Village, Ledong(M) | 108°42'36''N 18°40'48''E | June, 2023 | 30.8 | PQ200962 | PQ199364 |
| 11-8 | *Saccostrea malabonensis* | Haibin Village, Ledong(M) | 108°42'36''N 18°40'48''E | June, 2023 | 30.8 | PQ200955 |  |
| 11-9 | *Saccostrea echinata* | Haibin Village, Ledong(M) | 108°42'36''N 18°40'48''E | June, 2023 | 30.8 | PQ199976 | PQ199365 |
| 11-10 | *Saccostrea malabonensis* | Haibin Village, Ledong(M) | 108°42'36''N 18°40'48''E | June, 2023 | 30.8 | PQ200956 |  |
| 11-11 | *Saccostrea malabonensis* | Haibin Village, Ledong(M) | 108°42'36''N 18°40'48''E | June, 2023 | 30.8 | PQ199974 |  |
| 11-12 | *Saccostrea echinata* | Haibin Village, Ledong(M) | 108°42'36''N 18°40'48''E | June, 2023 | 30.8 | PQ200959 |  |
| 11-22 | *Saccostrea echinata* | Haibin Village, Ledong(M) | 108°42'36''N 18°40'48''E | June, 2023 | 30.8 | PQ200960 |  |
| 11-25 | *Saccostrea echinata* | Haibin Village, Ledong(M) | 108°42'36''N 18°40'48''E | June, 2023 | 30.8 | PQ199975 |  |
| 12-1 | *Saccostrea malabonensis* | Yulin Island Scenic Area, Dongfang(N) | 108°37'12''N 19°6'36''E | June, 2023 | 30.9 | PQ200969 | PQ199366 |
| 12-2 | *Saccostrea malabonensis* | Yulin Island Scenic Area, Dongfang(N) | 108°37'12''N 19°6'36''E | June, 2023 | 30.9 | PQ200970 |  |
| 12-3 | *Saccostrea echinata* | Yulin Island Scenic Area, Dongfang(N) | 108°37'12''N 19°6'36''E | June, 2023 | 30.9 | PQ200963 | PQ199380 |
| 12-4 | *Saccostrea echinata* | Yulin Island Scenic Area, Dongfang(N) | 108°37'12''N 19°6'36''E | June, 2023 | 30.9 | PQ200964 | PQ199367 |
| 12-5 | *Saccostrea malabonensis* | Yulin Island Scenic Area, Dongfang(N) | 108°37'12''N 19°6'36''E | June, 2023 | 30.9 | PQ200971 |  |
| 12-6 | *Crassostrea sikamea* | Yulin Island Scenic Area, Dongfang(N) | 108°37'12''N 19°6'36''E | June, 2023 | 30.9 | PQ200975 |  |
| 12-7 | *Saccostrea malabonensis* | Yulin Island Scenic Area, Dongfang(N) | 108°37'12''N 19°6'36''E | June, 2023 | 30.9 | PQ200972 |  |
| 12-8 | *Saccostrea malabonensis* | Yulin Island Scenic Area, Dongfang(N) | 108°37'12''N 19°6'36''E | June, 2023 | 30.9 | PQ200973 |  |
| 12-9 | *Saccostrea echinata* | Yulin Island Scenic Area, Dongfang(N) | 108°37'12''N 19°6'36''E | June, 2023 | 30.9 | PQ200965 |  |
| 12-10 | *Crassostrea sikamea* | Yulin Island Scenic Area, Dongfang(N) | 108°37'12''N 19°6'36''E | June, 2023 | 30.9 | PQ200976 | PQ199368 |
| 12-11 | *Crassostrea sikamea* | Yulin Island Scenic Area, Dongfang(N) | 108°37'12''N 19°6'36''E | June, 2023 | 30.9 | PQ200977 |  |
| 12-12 | *Saccostrea echinata* | Yulin Island Scenic Area, Dongfang(N) | 108°37'12''N 19°6'36''E | June, 2023 | 30.9 | PQ200966 |  |
| 12-13 | *Saccostrea malabonensis* | Yulin Island Scenic Area, Dongfang(N) | 108°37'12''N 19°6'36''E | June, 2023 | 30.9 | PQ200974 |  |
| 12-15 | *Saccostrea echinata* | Yulin Island Scenic Area, Dongfang(N) | 108°37'12''N 19°6'36''E | June, 2023 | 30.9 | PQ200967 |  |
| 12-16 | *Saccostrea echinata* | Yulin Island Scenic Area, Dongfang(N) | 108°37'12''N 19°6'36''E | June, 2023 | 30.9 | PQ200968 |  |
| 12-17 | *Crassostrea sikamea* | Yulin Island Scenic Area, Dongfang(N) | 108°37'12''N 19°6'36''E | June, 2023 | 30.9 | PQ200978 |  |
| 12-18 | *Crassostrea sikamea* | Yulin Island Scenic Area, Dongfang(N) | 108°37'12''N 19°6'36''E | June, 2023 | 30.9 | PQ200979 |  |
| 13-3 | *Dendostrea folium* | Leigong Island, Chengmai(X) | 109°52'48''N 19°59'24''E | June, 2023 | 29.3 | PQ200986 | PQ199369 |
| 13-6 | *Saccostrea cucullate* 2 | Leigong Island, Chengmai(X) | 109°52'48''N 19°59'24''E | June, 2023 | 29.3 | PQ200983 | PQ199382 |
| 13-14 | *Saccostrea malabonensis* | Leigong Island, Chengmai(X) | 109°52'48''N 19°59'24''E | June, 2023 | 29.3 | PQ200980 |  |
| 13-16 | *Saccostrea echinata* | Leigong Island, Chengmai(X) | 109°52'48''N 19°59'24''E | June, 2023 | 29.3 | PQ200988 |  |
| 13-25 | *Saccostrea malabonensis* | Leigong Island, Chengmai(X) | 109°52'48''N 19°59'24''E | June, 2023 | 29.3 | PQ200981 |  |
| 13-26 | *Saccostrea cuccullata* 3 | Leigong Island, Chengmai(X) | 109°52'48''N 19°59'24''E | June, 2023 | 29.3 | PQ200984 |  |
| 13-34 | *Dendostrea folium* | Leigong Island, Chengmai(X) | 109°52'48''N 19°59'24''E | June, 2023 | 29.3 | PQ200987 | PQ199371 |
| 13-35 | *Saccostrea mordax* 2 | Leigong Island, Chengmai(X) | 109°52'48''N 19°59'24''E | June, 2023 | 29.3 | PQ200985 |  |
| 13-40 | *Saccostrea malabonensis* | Leigong Island, Chengmai(X) | 109°52'48''N 19°59'24''E | June, 2023 | 29.3 | PQ200982 |  |
| 13-44 | *Saccostrea cuccullata* 3 | Leigong Island, Chengmai(X) | 109°52'48''N 19°59'24''E | May, 2024 | 29.3 | PQ200989 |  |
| 13-45 | *Saccostrea echinata* | Leigong Island, Chengmai(X) | 109°52'48''N 19°59'24''E | May, 2024 | 29.3 | PQ200990 |  |
| 13-46 | *Saccostrea echinata* | Leigong Island, Chengmai(X) | 109°52'48''N 19°59'24''E | May, 2024 | 29.3 | PQ200991 |  |
| 13-48 | *Saccostrea malabonensis* | Leigong Island, Chengmai(X) | 109°52'48''N 19°59'24''E | May, 2024 | 29.3 | PQ200992 |  |
| 13-50 | *Saccostrea cuccullata* 3 | Leigong Island, Chengmai(X) | 109°52'48''N 19°59'24''E | May, 2024 | 29.3 | PQ200993 |  |
| 13-51 | *Saccostrea malabonensis* | Leigong Island, Chengmai(X) | 109°52'48''N 19°59'24''E | May, 2024 | 29.3 | PQ200994 |  |
| 13-57 | *Saccostrea echinata* | Leigong Island, Chengmai(X) | 109°52'48''N 19°59'24''E | May, 2024 | 29.3 | PQ200995 |  |
| 13-58 | *Saccostrea cuccullata* 1 | Leigong Island, Chengmai(X) | 109°52'48''N 19°59'24''E | May, 2024 | 29.3 | PQ200996 | PQ199394 |
| 13-59 | *Saccostrea echinata* | Leigong Island, Chengmai(X) | 109°52'48''N 19°59'24''E | May, 2024 | 29.3 | PQ200997 |  |
| 13-60 | *Saccostrea cuccullata* 3 | Leigong Island, Chengmai(X) | 109°52'48''N 19°59'24''E | May, 2024 | 29.3 | PQ200998 | PQ199395 |
| 13-63 | *Saccostrea malabonensis* | Leigong Island, Chengmai(X) | 109°52'48''N 19°59'24''E | May, 2024 | 29.3 | PQ200999 |  |
